# Management of short-term recurrent obstructive jaundice after endoscopic ultrasound guided hepaticogastrostomy

**DOI:** 10.1055/a-2715-4429

**Published:** 2025-10-21

**Authors:** Wei Zhang, Lichao Zhang, Senlin Hou

**Affiliations:** 1 71213 Department of Biliopancreatic Endoscopic Surgery, The Second Hospital of Hebei Medical University, Shijiazhuang, Hebei, China

Endoscopic retrograde cholangiopancreatography (ERCP) is the preferred option for treating obstructive complications after digestive tract reconstruction surgery. However, endoscopic ultrasound-guided biliary drainage (EUS-BD) is a remedial measure when ERCP fails 1 2 . When patients experience a recurrence of obstructive jaundice after endoscopic ultrasound (EUS)-guided hepaticogastrostomy (HGS), there are not many treatment options available. In this article, we provide a solution for the short-term recurrence of obstructive jaundice after HGS, hoping that it can be useful to everyone.

Ten days ago, a middle-aged male patient who had undergone total gastrectomy was admitted for HGS treatment due to obstructive jaundice. Now, the patient has been readmitted due to the recurrence of obstructive jaundice. The patient refused PTCD. Considering that the fistula tract of HGS had not formed, we could not place the stent along the fistula. As the patient had hilar biliary obstruction, we decided to attempt the bridging technique to achieve bilateral drainage of the left and right hepatic ducts. We selected the puncture sites for the bile ducts in the S2 ( Fig. 1 **c** ) and S3 ( Fig. 1 **a** ) segments of the liver, attempted the bridging technique but failed, and respectively placed plastic stents with lengths of 9 cm ( Fig. 1 **d** ) and 10 cm ( Fig. 1 **b** ) and a diameter of 7 Fr. Finally, we attempted to perform a puncture in the S4 ( Fig. 1 **e** ) segment of the liver. We successfully inserted the guide wire into the bile duct of the right liver and placed a 12-cm, 7-Fr stent ( Fig. 1 **f** ) between the liver and the stomach, thus achieving the bridging technique. On the third day after the operation, the patientʼs bilirubin level improved significantly and was discharged smoothly ( Video 1 ).

**Fig. 1:**
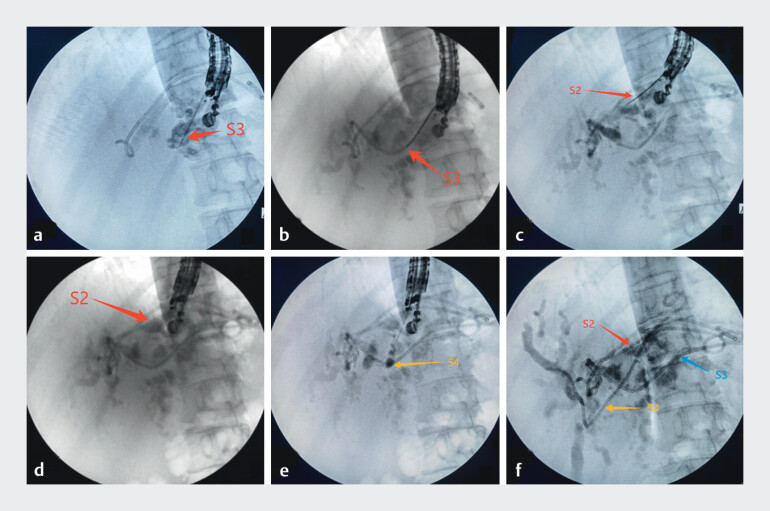
**a** The 19G puncture needle was inserted into the bile duct of the S3 segment of the liver. **b** A biliary stent was placed in the S3 segment of the bile duct. **c** The 19G puncture needle was once again inserted into the bile duct of the S2 segment of the liver. **d** A biliary stent was placed in the S2 segment of the bile duct. **e** The 19G puncture needle was inserted into the bile duct of the S4 segment of the liver. **f** The stent passes through the S4 segment of the bile duct and reaches the S8 segment to complete the bridging technique.

The puncture needle guided by endoscopic ultrasound was inserted into the target bile duct.Video 1

Previous studies have reported that the recurrence rate of obstructive jaundice after EUS-BD is 11– 25% 3 . Recurrence of obstructive jaundice within a short period is not very common. Our centerʼs previous experience is to replace the stent along the sinus tract or place multiple stents through the fistula tract 4 . This patient had recurrence of obstructive jaundice within a short period, so we could only choose to place a stent at a different puncture site or implement the bridging technique 5 . Implementing EUS-BD management for recurrent obstructive jaundice through multiple puncture sites requires sufficient experience and strict indications. We hope that this experience can provide a reference for dealing with recurrence of obstructive jaundice after EUS-BD.

Endoscopy_UCTN_Code_TTT_1AS_2AH

## Notes

Endoscopy E-Videos https://eref.thieme.de/e-videos
*E-Videos* is an open access online section of the journal *Endoscopy* , reporting on interesting cases and new techniques in gastroenterological endoscopy. All papers include a high-quality video and are published with a Creative Commons CC-BY license. Endoscopy E-Videos qualify for HINARI discounts and waivers and eligibility is automatically checked during the submission process. We grant 100% waivers to articles whose corresponding authors are based in Group A countries and 50% waivers to those who are based in Group B countries as classified by Research4Life (see: https://www.research4life.org/access/eligibility/ ). This section has its own submission website at https://mc.manuscriptcentral.com/e-videos .
